# Radiopacity of Methacrylate and Silorane Composite Resins Using a Digital Radiographic System

**DOI:** 10.1155/2016/6389347

**Published:** 2016-09-19

**Authors:** Leily Macedo Firoozmand, Mariana Gonçalves Cordeiro, Marcos André dos Santos Da Silva, Rudys Rodolfo De Jesus Tavarez, Etevaldo Matos Maia Filho

**Affiliations:** ^1^Department of Restorative Dentistry, Universidade Federal do Maranhão (UFMA), School of Dentistry, São Luís, MA, Brazil; ^2^Universidade Ceuma (UniCeuma), School of Dentistry, Graduate Program in Dentistry, São Luís, MA, Brazil; ^3^Department of Radiology, Universidade Ceuma (UniCeuma), School of Dentistry, São Luís, MA, Brazil; ^4^Department of Restorative Dentistry, Universidade Ceuma (UniCeuma), School of Dentistry, São Luís, MA, Brazil

## Abstract

The aim of this study was to evaluate the radiopacity of silorane and methacrylate resin composites, comparing them to the enamel, dentin, and aluminum penetrometer using a digital image. From six resin composites (Filtek*™* P90, Filtek Z350, Filtek Z350 XT flow, Tetric Ceram, TPH Spectrum, and SureFil SDR flow) cylindrical disks (5 × 1 mm) were made and radiographed by a digital method, together with a 15-step aluminum step-wedge and a 1 mm slice of human tooth. The degree of radiopacity of each image was quantified using digital image processing. The mean values of the shades of gray of the tested materials were measured and the equivalent width of aluminum was calculated for each resin. The results of our work yielded the following radiopacity values, given here in descending order: Tetric Ceram > TPH > SDR > Z350 > Z350 flow > P90 > enamel > dentin. The radiopacity of the materials was different both for the enamel and for the dentin, except for resin P90, which was no different than enamel. In conclusion, silorane-based resin exhibited a radiopacity higher than dentin and closest to the enamel; a large portion of the methacrylate-based flow and conventional resins demonstrated greater radiopacity in comparison to dentin and enamel.

## 1. Introduction

Resin composites are restorative materials that have been gaining wide acceptance due to their broad clinical applications. With advances in resin composites and bonding systems, a large portion of restorations are currently performed with these materials on both posterior and anterior teeth [1]. However, certain principles must be followed properly to ensure the longevity of the restoration and to avoid the occurrence of postoperative sensitivity, microleakage, and the return of caries [1]. Dental materials should have sufficient radiopacity in relation to dentin and enamel to allow the proper evaluation of the margins between the restorative material and dental substrate and the visualization of the contour of the restoration, contact with the adjacent dental tissue, marginal defects, and the detection of secondary caries [2–4].

On posterior teeth, resin composites should be at least as radiopaque as enamel [5]. When these materials are insufficiently radiopaque, it is more difficult to view microleakage, pits, fissures, and early carious lesions on radiographs [3].

Besides the conventional (medium and high viscosity) resin composites that are recommended for the restoration on posterior teeth, some low viscosity resins (flow materials) may also be indicated for the restoration of posterior teeth. Flow resins, which have a low particle density, function as a “shock absorber” during occlusal impacts due to their lower modulus of elasticity [1], offering less of a challenge to the adhesive integrity of restorations on posterior teeth.

A large portion of current resin composites include methacrylate, the polymeric chain of which is formed by the replacement of van der Waals spaces with shorter covalent bonds [6], causing the contraction of the material during polymerization [1]. In response to this drawback, a silorane-based organic resin matrix has been created as an alternative to methacrylate-based systems [7]. The two main advantages of this material are the lower contraction during polymerization due to the opening of the ring of the oxirane monomer and increased hydrophobia due to the presence of siloxane [8].

Silorane-based resins were introduced in a category of a material commercially marketed as a low-shrinkage composite that should be better investigated in both the clinical and laboratory settings. While some laboratory studies [9] show that silorane-based resins have low bond strength and greater nanoleakage expression of these resins compared to methacrylate-based restorations, clinical evaluations of these resins seem to demonstrate stable proximal contact both immediately following class II restorations and after six months of clinical follow-up [10].

Digital radiography has gradually been replacing conventional radiographs. The implementation of digital images in dental offices is a time-saving measure that allows the manipulation of images [11]. This radiographic analysis method is easy, fast, and reliable [12]. Moreover, studies have shown that digital radiography provides a precise diagnosis similar to that achieved with conventional radiography [13, 14].

Resin composites have different degrees of radiopacity [2, 15] and investigations need to be conducted on the new materials available on the market. Thus, the aim of the present study was to analyze the degree of radiopacity of methacrylate (flow and conventional) and silorane resins using a digital radiographic system. The null hypothesis is that no significant differences are found in the radiopacity of the methacrylate and silorane resin composites analyzed and that no significant differences are found between dentin and enamel.

## 2. Methodology

This study was approved by the research ethics committee of Ceuma University (Protocol number 1378157).

Samples measuring 5 mm in diameter and 1 mm in thickness were formed using a cylindrical matrix. Five samples of each material displayed in Table 1 were made following the manufacturers' instructions. The resins were inserted into the matrix in a single portion with the aid of a nonadherent spatula. A glass slide was positioned with light pressure over the matrix and care was taken to obtain a flat, uniform surface free of air bubbles.

The samples were photoactivated for 40 s with an Optilux 501 (Kerr/Demetron, Orange, CA, USA) under a light intensity of 600 mW/cm^2^ and were measured with a calibrated power meter (ECEL-RD, Dabi Atlante, São Paulo, Brazil). After removal from the cylindrical matrix, the samples were measured using a digital caliper to determine the thickness, for which the tolerance was 1 ± 0.01 mm. The samples were then stored in distilled water for 24 h and submitted to radiographic analysis. For the purposes of comparison, a human molar extracted for orthodontic reasons was used to obtain a cross-section (enamel, dentin, and pulp) measuring 1 mm in thickness made using a diamond cutting disk (St. Joseph, MI, USA) operating at 250–300 rpm.

A positioning device was used to place the samples at a standardized focal distance (30 cm). To obtain the digital images, the SPECTRO-70X® dental X-ray device (Dabi Atlante, São Paulo, SP, Brazil) was used with the following parameters: 10 mA, 65 kVp, and 0.1 s exposure time. The XIOS CMOS Intraoral Digital System (Sirona, Bensheim, Germany) was coupled to the X-ray device.

The slice of human tooth, six composite resin samples (one from each group), and a 15-step aluminum step-wedge, made of 99.5% pure aluminum with seven 1 mm thick incremental steps, were placed on the radiographic sensor.

**Figure 1:**
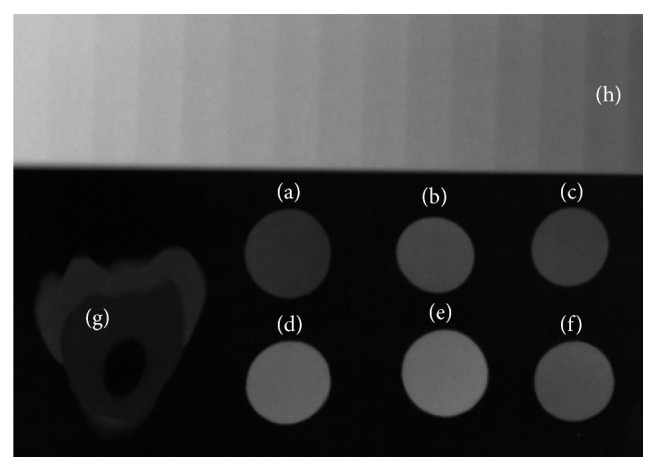
Radiographic image of resin samples ((a) P90, (b) Z350, (c) Z350 flow, (d) Tetric Ceram, (e) TPH, (f) SDR), slice of human tooth (g), and aluminum step-wedge (h).

All radiographic images (Figure 1) were evaluated by the same examiner, using the SIDEXIS XG software program (Sirona, Bensheim, Germany). The examiner had previously undergone training, and calibration was done by twice measuring five radiographic images, with a 2-week interval between the first and second measurements. The interrater degree of agreement was calculated using the Intraclass Correlation test (ICC = 0.968).

Radiopacity was measured in three different regions of each sample. Care was taken to make readings only of regions without air bubbles, gaps, or other defects. The same procedure was performed on different regions of the tooth sample.

Mean shade of gray values for each resin, aluminum step-wedge, and enamel and dentin were measured using the Image J software application (National Institutes of Health, USA). Radiopacity, on a shade of gray scale, was converted into the equivalent of millimeters of aluminum (mm Al) for all the materials tested. To this end, the radiopacity was measured at each step of the aluminum scale (aluminum step-wedge) on the radiograph. A graph was constructed of the radiopacity versus the thickness of each step on the scale. A regression equation was obtained, *Y* = *a* + *bX*, where *Y* is the radiopacity, *a* is the regression constant, *b* is the slope of the line, and *X* is the thickness of the step on the scale. The equivalent to aluminum thickness for each resin (*X*) was calculated through the equation [(*Y* − *a*)/*b*].

## 3. Statistical Analysis

The Shapiro-Wilk test was used to determine the distribution of the data. Given the normal distribution of data (*p* > 0.05), by means of a one-way ANOVA test and Tukey's post hoc test, an evaluation was made as to whether there was a significant difference in radiopacity between the resins. In addition, the one-way ANOVA test with Dunnett's post hoc was applied in order to test the hypothesis of whether there was a significant difference between the radiopacity values of the resins and the radiopacity of the dentin and enamel.

Using the one-sample Student* t*-test, an evaluation was made as to whether the mean radiopacity values of the resins, expressed in mm Al, were other than 1 mm Al. The level of significance was set to 5% (*p* < 0.05). Data analysis was performed using the IBM SPSS Statistics 21.0 (Armonk, New York, USA).

## 4. Results

Table 2 shows the mean values (±standard deviation) of the radiopacity and the equivalent mm Al value of the materials evaluated. The radiopacity of the respective materials, in descending order, is as follows: Tetric Ceram > TPH > SDR > Z350 > Z350 flow > P90 > enamel > dentin.

A one-way ANOVA test showed that there was a significant difference in radiopacity between the resins (*p* < 0.05). A two-by-two comparison (Tukey's post hoc) can be seen in Table 2.

A one-way ANOVA test with Dunnett's post hoc showed that the radiopacity of all the materials was different for both enamel (*p* < 0.05) and for dentin (*p* < 0.05), except for resin P90, which did not exhibit any significant difference when compared with enamel (*p* > 0.05).

With the exception of the P90 resin, all the resins presented significant differences from the aluminum 1 mm (*p* < 0.05).

## 5. Discussion

The study of the radiopacity of direct restorative materials has considerable importance, as the use of radiolucent materials or those with similar radiopacity to dentin can mask microleakage and secondary carious lesions [2, 4], leading to diagnostic errors. The analysis of the resin composites tested herein demonstrated that the null hypothesis was rejected.

According to the recommendations of the International Organization for Standardization (ISO 40490:2009) [16] restoration materials measuring 1 mm in thickness should have radiopacity equal to or greater than the same thickness of aluminum. According to results found in the literature [12] all tested materials followed the ISO 4049 standard and showed radiopacity value greater than a 1 mm step of the penetrometer scale, because it is known that the radiopacity material may vary according to the material thickness [17]. In the present study 10 mA, 65 kVp and 0.1 s were applied. It is observed that values of absorbance of the aluminum step-wedge vary in the literature, because different combinations of voltage and exposure are employed [12].

The composition of the composite resin has great influence on the radiopacity of the material [17]. To increase the radiopacity of dental restoration materials, elements with a high atomic number, such as barium, strontium, zirconia, and zinc, have been added to the inorganic phase of resins with no adverse effects on the other properties of these materials. In the present study, the silorane-based resin (P90), which has a low degree of contraction during polymerization, was the most radiolucent material tested due to the small amount of radiopaque elements in its composition. But the results of this study showed that the P90 composite resin, which is indicated for the restoration of anterior and posterior teeth, demonstrated radiopacity greater than dentin and similar to the dental enamel.

Tetric Ceram (microhybrid composite resin) demonstrated the greatest degree of radiopacity, followed by TPH Spectrum (microhybrid composite resin). Regarding the flow materials (low viscosity) used in this study, both low polymerization shrinkage (Surefil SDR flow) and conventional resin (FIltek Z350 flow) presented similar radiopacity to the high viscosity composite resin Filtek Z350, which contains nanoparticles. According to the manufacturer, SureFil SDR contains radiopaque components, giving it satisfactory radiopacity based on the criteria established in the literature [5, 17]. The flow resin composites are made with inorganic particles of the same size as those in resin composites, but with lower compactness, resulting in lower viscosity [1]. Interestingly, the results of this study showed the majority of the high viscosity resin composites presented higher radiopacity levels than the flowable resin composites [18]. The important point is that these materials demonstrated greater radiopacity in comparison to dental enamel and dentin.

Radiopacity is an essential property for restorations on posterior teeth. However, there are a number of resins indicated to restore anterior and posterior teeth that have insufficient radiopacity for adequate treatment [19]. In the present study, the different resin composites demonstrated different degrees of radiopacity, which is in agreement with data reported in the literature [18–20]. A large portion of resin composites demonstrate radiopacity equal to or greater than tooth enamel [15], whereas others demonstrate radiopacity equal to dentin or between dentin and enamel. Thus, it is of extreme importance to evaluate the characteristics of new materials launched on the market to ensure that such products can provide lasting restorations.

## 6. Conclusions

Most of the tested composites have higher radiopacity than the dentin and enamel. The resin-based silorane P90 showed radiopacity closest to tooth enamel, while the methacrylate-based resin showed the highest degree of radiopacity.

## Figures and Tables

**Table 1:** Materials and characteristics.

Group	Composite resin	Commercial brand	Composition	Lot
G1	Filtek P90	3M/ESPE, St. Paul, MN, USA	Microhybrid Quartz and radiopaque yttrium fluoride (Silorane)	N322267

G2	Filtek Z350	3M/ESPE, St. Paul, MN, USA	Nanoparticles and nanoclusters Silica/zirconia (Bis-GMA/TEGDMA)	N355898

G3	Filtek Z350 XT flow	3M/ESPE, St. Paul, MN, USA	Nanoparticles and nanoclusters Silica/zirconia (Bis-GMA/TEGDMA)	N399586

G4	Tetric Ceram	Ivoclar-Vivadent, Schaan, Liechtenstein	Microhybrid Barium glass, barium fluorosilicate-aluminum glass, ytterbium trifluoride, silicon dioxide, and oxides (Bis-GMA/TEGDMA)	P87674

G5	TPH Spectrum	Dentsply Caulk, Milford, DE, USA	Microhybrid Silanized aluminum boron silicate, barium, and pyrolytic silica (Bis-GMA/TEGDMA)	720359E

G6	SureFil SDR flow	Dentsply Caulk, Milford, DE, USA	(Bis-GMA/TEGDMA) Size not informed by manufacturer Aluminum silicate barium fluoride glass, and strontium aluminum silicate fluoride glass	671513E

**Table 2:** Radiopacity (shades of gray) and the aluminum equivalent in millimeters (mm Al) of the different materials evaluated.

	Mean gray	Radiopacity value (mm Al equivalent)

Tetric Ceram	84.20 (±9.68)^A^	3.10 (±0.32)^x,y^
TPH	72.20 (±7.05)^B^	2.65 (±0.31)^x,y^
SDR	52.60 (±3.51)^CD^	1.94 (±0.14)^x,y^
Z350	48.20 (±2.77)^DE^	1.78 (±0.12)^x,y^
Z350 flow	38.40 (±3.97)^E^	1.44 (±0.16)^x,y^
P90^*∗*^	25.60 (±1.00)^F^	0.99 (±0.12)^y^

Enamel	22.60 (±5.86)	0.91 (±0.22)
Dentin	14.20 (±2.28)	0.63 (±0.12)

Different letters: significant difference between resins, *p* < 0.05 (ANOVA-Tukey test).

Significant difference for the enamel (x) and dentin (y), *p* < 0.05 (ANOVA-Dunnett's test).

^*∗*^No significant difference for the 1 mm Al, *p* > 0.05 (one-sample *t*-test).
